# An Overproduction of Scholars

**Published:** 1893-01

**Authors:** 


AN OVERPRODUCTION OF SCHOLARS.—In an article in the "Forum" for January by Dr. Geffcken the curious fact is brought out that in Germany there has been too much of the higher education. Special culture, unsustained by an income from property, is found to be in many cases a misfortune. The average demand in Prussia for "assessors"—men of legal training who have passed through the gymnasium and university and served the State gratis for five years—is 100, while the actual number is, 1,851. There is thus no employment for very many of those prepared every year for the legal profession. There are over 7,000 examined architects without fixed employment. It is the same with engineers, teachers in the classics, mathematics, &c. "Men of university training," says Dr. Geffcken, "are almost without exception capable only of intellectual work." They cannot, or will not, take to a trade, but flock to the cities and take to pettifogging, giving lessons, writing for inferior papers, &c. "There are lawyers, physicians and doctors of philosophy," he adds, "among those who are regularly relieved by the Berlin poor board." It was once believed that the State had no more important duty than to educate the greatest possible number of its youths to the greatest possible extent. This, it seems, is a mistake. Germany's educational system is top-heavy. There are perhaps other countries where the supply of men educated for the professions is excessive. The world's work is not all head work. Different communities require it in different quantities, according to their varied circumstances.

